# Process and outcome indicators for infection control and prevention in European acute care hospitals in 2011 to 2012 – Results of the PROHIBIT study

**DOI:** 10.2807/1560-7917.ES.2018.23.21.1700513

**Published:** 2018-05-24

**Authors:** Sonja Hansen, Frank Schwab, Walter Zingg, Petra Gastmeier, the PROHIBIT study group

**Affiliations:** 1 Charité - Universitätsmedizin Berlin, corporate member of Freie Universität Berlin, Humboldt-Universität zu Berlin, and Berlin Institute of Health, Institute for Hygiene and Environmental Medicine, Berlin, Germany; 2 University of Geneva Hospitals, Infection Control Programme, Switzerland; 3 The members of the PROHIBIT study group are listed at the end of the article

**Keywords:** Infection control and prevention, healthcare-associated infections, multidrug-resistant organisms, surveillance, acute-care hospitals, Europe

## Abstract

Hospitals from 24 European countries were asked for information on infection prevention and control (IPC) indicators as part of the Prevention of Hospital Infections by Intervention and Training (PROHIBIT) survey. **Methods:** Leading IPC personnel of 297 hospitals with established healthcare-associated infection (HCAI) surveillance provided information on local surveillance and feedback by using a questionnaire. **Results:** Most hospitals focused on bloodstream infection (BSI) (n = 251) and surgical site infection (SSI) (n = 254), with a SSI post-discharge surveillance in 148 hospitals. As part of the HCAI surveillance, meticillin-resistant *Staphylococcus aureus* (MRSA) was the leading multidrug-resistant organism (MDRO) under surveillance. Seventy-nine per cent of hospitals (n = 236) monitored alcohol-based hand rub (ABHR) consumption. Feedback to the local IPC committees mainly included outcome data on HCAI (n = 259; 87%) and MDRO among HCAI (n = 245; 83%); whereupon a feedback of MDRO data depended on hospital size (p = 0.012). **Discussion/conclusion:** Objectives and methods of surveillance vary across Europe, with BSI, SSI and MRSA receiving considerably more attention than indicators such as pneumonia and urinary tract infection, which may be equally important. In order to maximise prevention and control of HCAI and MDRO in Europe, surveillance should be further improved by targeting relevant HCAI. The role of feedback should be explored in more detail.

## Introduction

Based on the results of the first European point prevalence survey (PPS) in 2011–12 an estimated 3.2 million patients acquire a healthcare-associated infection (HCAI) in acute care hospitals in Europe every year [1]. The most common types of HCAI are surgical site infections (SSI), urinary tract infections (UTI), pneumonia (PN), bloodstream infections (BSI), and gastrointestinal infections, with *Clostridium difficile* infection (CDI) accounting for a high proportion in the latter. HCAIs result in increased morbidity and mortality, and emerging antibiotic resistance complicates their treatment. The cumulative burden of HCAIs is higher than the total burden of other communicable diseases in Europe [2].

Surveillance as the ‘ongoing systematic collection and analysis of health data for the planning, implementation, and evaluation, of public health practice’ [3] is a key measure in HCAI prevention and control. Even in the absence of specific prevention actions, surveillance and feedback of outcome indicators decrease HCAI by raising awareness for the issue among healthcare professionals [4-7]. Surveillance, preferably as part of a network, was identified as one of the key components in effective HCAI prevention and an important tool for monitoring the effectiveness of prevention and control measures by the ‘Systematic Review and Evidence-based Guidance on Organization of Hospital Infection Control Programmes’ (SIGHT) project [8].

Since the 1990s, many European countries have been developing national surveillance networks, either by applying the United States’ (US) Centers for Disease Control and Prevention (CDC) National Nosocomial Infection Surveillance/National Healthcare Safety Network (NNIS/NHSN) protocol, or by using adapted methods to better take into account local diagnostic practices [9]. In 2010, the European Centre for Disease Prevention and Control (ECDC) established the Healthcare-Associated Infections surveillance Network (HAI-Net), integrating the Hospitals in Europe Link for Infection Control through Surveillance (HELICS) project (2000–4) and the Improving Patient Safety in Europe (IPSE) network (2005–8) [10].

Surveillance of alcohol-based hand rub (ABHR) consumption has become a mandatory quality indicator with public reporting in France since 2006 [11], and was integrated into the national Krankenhaus-Infektions-Surveillance-System (KISS) in 2008 in Germany [12]. Furthermore, national strategies on measuring hand hygiene compliance by direct observation have been organised in a number of European countries [13].

Aspects of specific surveillance activities in European hospitals were obtained as part of the Prevention of Hospital Infections by Intervention and Training (PROHIBIT) project. The PROHIBIT survey was conducted as the first pan-European survey on infection prevention and control (IPC) in order to describe which IPC recommendations are actually being used across Europe and to provide information on gaps in hospitals’ IPC policies and practices for policymakers, hospital managers and healthcare workers for further improvement of HCAI prevention. This article summarises data on findings from 24 European countries.

## Methods

### Participating countries and hospitals

ECDC national contact points (NCPs) and IPC experts of European countries outside of the European Union (EU) were invited to organise national polls. The NCPs invited national hospitals for participation between September 2011 and March 2012. Participation in the PROHIBIT survey was based mainly on hospital interest rather than on a systematic sampling process.

### Survey description

The survey was developed by an interdisciplinary group and discussed with the HAI-Net representatives. It included four questionnaires in order to assess IPC structure and process indicators (i) at the hospital level, (ii) in intensive care units (ICU), (iii) in non-ICU medical wards and (iv) in non-ICU surgical wards. Questionnaires addressed organisation and activities of IPC at those various levels.

The complete method of the survey and the characteristics of the participating hospitals are described in more detail elsewhere [14].

For the present analysis, data on HCAI surveillance, process and outcome indicators (e.g. ABHR consumption, HCAI), direct hand hygiene observations, feedback practices, and persons performing surveillance are described at hospital level. Hospitals were asked whether the following multidrug-resistant organisms (MDRO) were monitored among HCAI: meticillin-resistant *Staphylococcus aureus* (MRSA), vancomycin-resistant Enterococci (VRE), extended-spectrum beta-lactamase (ESBL)-producing Enterobacteriaceae, carbapenem nonsusceptible or carbapenemase-producing Enterobacteriaceae, carbapenem-resistant *Pseudomonas aeruginosa* and multiresistant *Acinetobacter baumannii.*

Local IPC professionals were also asked to provide data on hospital characteristics such as status (public/private) and size of the hospital (number of beds), and the full time equivalent (FTE) of infection control personnel.

Furthermore, country characteristics such as the United Nations (UN) European geographical region, and healthcare expenditure (HCE) as share on the national gross domestic product (GDP) were collected [15,16].

### Data analysis

Descriptive data analysis was performed and results summarised as totals and strata of the following four parameters: hospital size (small: ≤ 300 beds; medium: 301–600 beds; large: > 600 beds), UN European geographical regions, full-time-equivalent (FTE) infection control nurses (ICN) (internal staff and external staff) per 1,000 acute care hospital beds ≤ / > the median of all participating hospitals (3.72 FTE ICN/1,000 beds) and HCE as share on GDP [15,16]. HCE was modelled as a dichotomous variable and considered low or high if below or above the European mean HCE of 9%. Differences in the process and outcome indicators between the strata of the four parameters described above were tested by logistic regressions models. In the regression analysis with indicator parameters as outcome, only the independent variables were included in the generalised estimating equation (GEE) models, adjusting for cluster effects by country. A two-sided p value < 0.05 in the type III test was considered significant. All analyses were performed with SPSS (IBM SPSS statistics, Somer, NY, US) and SAS (SAS Institute, Cary, NC, US).

## Results

### Participating countries and hospitals

Of 32 invited countries, 24 participated (Table 1) with 309 acute care hospitals. From all 309 acute care hospitals participating in the PROHIBIT survey, 297 hospitals (96%) had some method of HCAI surveillance in place. Hospitals with HCAI surveillance had a median of 426 beds (interquartile range (IQR): 260–277), and were most often public hospitals (253 hospitals, 85%).

**Table 1:** Distribution of hospitals providing data on healthcare-associated infection surveillance and national healthcare expenditure as part of the gross domestic product by country – the Prevention of Hospital Infection by Intervention and Training (PROHIBIT) survey, Europe, 2011–2012 (n = 297 hospitals)

United Nations region^a^	Country	Total HCE as % of GDP^b^	Number of participating hospitals	
			n	%
Northern Europe, n = 70	Finland	8.9	11	3.7
	Ireland	9.2	12	4
	Latvia	6.8	7	2.4
	Lithuania	7	13	4.4
	Sweden	9.6	6	2
	United Kingdom, England	9.6	5	1.7
	United Kingdom, Scotland		3	1
	United Kingdom, Wales		13	4.3
Eastern Europe, n = 82	Bulgaria	7.2	19	6.4
	Hungary	7.8	30	10.1
	Poland	7	9	3
	Slovakia	9	24	8.1
Southern Europe, n = 81	Croatia	7.8	5	1.7
	Italy	9.3	18	6.1
	Malta	8.6	1	0.3
	Portugal	10.7	26	8.8
	Slovenia	9	8	2.7
	Spain	9.6	23	7.7
Western Europe, n = 64	Austria	11	8	2.7
	Belgium	10.5	5	1.7
	France	11.6	8	2.7
	Germany	11.6	29	9.8
	Switzerland	11.4	6	2
	The Netherlands	12	8	2.7
All	NA	NA	297	100

GDP: gross domestic product; HCE: healthcare expenditure; NA: not applicable.

**^a ^**Geographical regions according to United Nations grouping [15].

**^b ^**HCE as the share of the GDP [16].

### Medical conditions/disease outcome where HCAI surveillance applies

Surveillance in the hospitals mainly focused on SSI and BSI, and less often on PN, CDI and UTI (Table 2). Surveillance of UTI depended on countries’ HCE with significantly higher proportions in countries with low HCE. For PN, significant differences were observed in accordance to the hospital size, with more medium to large hospitals having PN surveillance in place compared to smaller hospitals. Significantly more hospitals from countries with low HCE performed hospital-wide surveillance of PN and UTI. Hospital-wide surveillance of BSI varied significantly with the UN regions. 

**Table 2:** Surveillance of process and outcome indicators in European acute care hospitals, stratified by healthcare expenditure, United Nation regions, hospital size, rate of infection control nurses – the Prevention of Hospital Infection by Intervention and Training (PROHIBIT) survey, 2011–2012 (n = 297 hospitals)

Healthcare-associated infections under surveillance with particular procedures surveilled	Total		HCE^a^				United Nations region^b^								Hospital size^c^						ICN/1,000 beds^d^			
			Low		High		Eastern Europe		Northern Europe		Southern Europe		Western Europe		≤ 300 beds		301–600 beds		> 600 beds		≤ median		> median	
	N	%	N	%	N	%	N	%	N	%	N	%	N	%	N	%	N	%	N	%	N	%	N	%
	297		127		170		82		70		81		64		87		109		98		150		147	
Healthcare-associated infections under surveillance																								
Bloodstream infections	251	85	112	88	139	82	79	96	58	83	65	80	49	77	67	77	96	88	85	87	127	85	124	84
• Hospital-wide^e,f^	175	59	91	72	84	49	68	83	49	70	42	52	16	25	50	57	72	66	51	52	79	53	96	65
Pneumonia^g^	211	71	103	81	108	64	74	90	39	56	56	69	42	66	49	56	84	77	76	78	113	75	98	67
• Hospital-wide^e,h^	97	33	67	53	30	18	49	60	20	29	22	27	6	9	26	30	42	39	29	30	48	32	49	33
Urinary tract infections^h^	187	63	101	80	86	51	74	90	28	40	49	60	36	56	49	56	69	63	68	69	103	69	84	57
• Hospital-wide^e,h^	109	37	72	57	37	22	54	66	22	31	27	33	6	9	31	36	47	43	31	32	52	35	57	39
*Clostridium difficile*-associated infections	203	68	76	60	127	75	50	61	54	77	55	68	44	69	51	59	73	67	76	78	91	61	112	76
• Hospital-wide^e^	191	64	71	56	120	71	45	55	54	77	51	63	41	64	46	53	69	63	73	74	86	57	105	71
SSI	254	86	112	88	142	84	75	91	63	90	62	77	54	84	68	78	92	84	91	93	129	86	125	85
Procedures																								
Cholecystectomy	130	44	71	56	59	35	48	59	22	31	33	41	27	42	31	36	57	52	42	43	71	47	59	40
Colon surgery	129	43	56	44	73	43	39	48	19	27	43	53	28	44	30	34	54	50	44	45	64	43	65	44
Caesarean section	126	42	63	50	63	37	45	55	35	50	23	28	23	36	26	30	54	50	44	45	62	41	64	44
Hip prosthesis implantation	164	55	57	45	107	63	32	39	48	69	48	59	36	56	40	46	65	60	56	57	75	50	89	61
Knee prosthesis implantation	138	46	42	33	96	55	24	29	41	59	43	53	30	47	33	38	57	52	45	46	57	38	81	55
Post discharge surveillance of SSI	148	50	54	43	94	55	35	43	40	57	43	53	30	47	39	45	59	54	48	49	64	43	84	57
Monitoring of alcohol-based handrub consumption	236	79	105	83	131	77	65	79	47	67	71	88	53	83	68	78	82	75	85	87	124	83	112	76
• Hospital-wide^e^	215	72	95	75	120	71	57	70	46	66	65	80	47	73	59	68	80	73	75	77	114	76	101	69
Monitoring of hand hygiene compliance^f^	231	78	101	80	130	76	62	76	67	96	66	81	36	56	72	83	81	74	75	77	109	73	122	83
• Hospital-wide^e,c^	173	58	79	62	94	55	48	59	62	89	38	47	25	39	59	68	64	59	49	50	79	53	94	64

FTE: full time equivalent; HCE: healthcare expenditure; ICN: infection control nurse; SSI: surgical site infection.

P values were calculated by logistic regression using generalised estimating equations, which account for cluster effects by country.

^a ^Low/high HCE defined as the share of the gross domestic product ≤ / > the European mean in 2010 (9%) [16].

^b ^Geographical regions according to United Nations grouping [15]; Eastern Europe, Northern Europe, Southern Europe, Western Europe.

^c ^Hospital size according to number of acute care beds; ≤ 300 beds, 301 – 600 beds, > 600 beds; information available for 294 hospitals.

^d ^FTE ICN (internal staff and external staff) per 1,000 acute care hospital beds ≤ / > the median of all participating hospitals (3.72 FTE ICN/1,000 beds).

^e^ Hospital-wide describes that the surveillance takes place in all units and wards of the hospital.

^f ^Differences between United Nations regions p < 0.05*.*

^g ^Differences between ≤ 300 beds / 301 – 600 beds / > 600 beds; p < 0.05*.*

^h ^Differences between low/high HCE; p < 0.05*.*

Surveillance of CDI was reported most often by hospitals in Northern Europe and more often by hospitals in countries with high HCE; but these differences were not statistically significant. 

Hip prosthesis implantation (HPRO) was the most common indicator operation of SSI surveillance with higher percentages in countries with high HCE; but these differences were not statistically significant. In 148 of 254 hospitals with SSI surveillance (58%), post-discharge surveillance (PDS) was in place.

### Multidrug-resistant organisms surveyed among HCAIs

MRSA was the most commonly observed MDRO among HCAI in almost all hospitals (n = 273), followed by ESBL-producing Enterobacteriaceae**(n = 243) and VRE (n = 228). Multidrug-resistant *Acinetobacter baumannii* surveillance was reported by 204 hospitals, carbapenem nonsusceptible or carbapenemase-producing Enterobacteriaceae by 189 hospitals, and carbapenem-resistant *Pseudomonas aeruginosa* by 185 hospitals.

### Monitoring hand hygiene compliance

Consumption of ABHR and hand hygiene compliance was observed in 79% and 78% of the hospitals, respectively (Table 2). Hospitals in countries in Northern Europe preferred monitoring hand hygiene compliance to monitoring ABHR consumption, while hospitals in countries in Western Europe preferred monitoring ABHR consumption to monitoring hand hygiene compliance (Table 2).

### Operators involved in HCAI surveillance

Table 3 summarises the operators involved in HCAI surveillance. Most surveillance activities are performed by IPC personnel. Although the overall numbers are low, it appeared that hospitals in countries of Northern Europe had a higher percentage of specific staff dedicated to surveillance of HCAI compared to other regions. In almost half of the hospitals, data on HCAI were collected by IPC personnel, whereas surveillance exclusively performed by ward personnel was by reported by 9% of all hospitals, and 18% of hospitals in Eastern Europe.

**Table 3:** Personnel involved in data collection for healthcare-associated infection (HCAI) surveillance in European acute care hospitals with HCAI surveillance, stratified by healthcare expenditure, United Nation regions, hospital size and infection control nurse rate – the Prevention of Hospital Infection by Intervention and Training (PROHIBIT) survey, 2011–2012 (n = 297 hospitals)

Personnel involved in data collection for HCAI surveillance	Total		HCE^a^				United Nation region^b^								Hospital size^c^ in terms of number of beds						ICN/1,000 beds^d^			
			Low		High		Eastern		Northern		Southern		Western		≤ 300		301–600		> 600		≤ median		> median	
	n = 297	%	n = 127	%	n = 170	%	n = 82	%	n = 70	%	n = 81	%	n = 64	%	n = 87	%	n = 109	%	n = 98	%	n = 150	%	n = 147	%
ICN^e^	217	73	70	55	147	86	45	55	50	71	65	80	57	89	62	71	79	72	73	74	99	66	118	80
Infection control physician^f^	146	49	54	43	92	54	34	41	36	51	51	63	25	39	32	37	54	50	58	59	79	53	67	46
Ward nurse	61	21	31	24	30	18	21	26	22	31	13	16	5	8	19	22	28	26	13	13	28	19	33	22
Ward physician	97	33	39	31	58	34	26	32	29	41	25	31	17	27	31	36	39	36	26	27	44	29	53	36
Specific surveillance staff e.g. nurse or administrator	9	3	5	4	4	2	5	6	2	3	2	2	0	0	1	1	4	4	3	3	6	4	3	2
Audit nurse	46	15	22	17	24	14	13	16	18	26	9	11	6	9	10	11	16	15	19	19	23	15	23	16
Infection control personnel exclusively (ICN and/or infection control physician)	143	48	56	44	87	51	36	44	24	34	43	53	40	63	39	45	49	45	53	54	76	51	67	46
Ward personnel exclusively (ward nurse and/or ward physician)	27	9	19	15	8	5	15	18	7	10	4	5	1	2	13	15	12	11	2	2	14	9	13	9
Infection control personnel and ward personnel	97	33	38	30	59	35	24	29	29	41	27	33	17	27	28	32	38	35	30	31	45	30	52	35

FTE: full time equivalent; GDP: gross domestic product; HCE: healthcare expenditure; ICN: infection control nurse.

P values were calculated by logistic regression using generalised estimating equations, which account for cluster effects by country.

^a ^Low/high HCE defined as the share of the GDP ≤ / > the European mean in 2010 (9%) [16].

^b ^Geographical regions according to United Nations grouping [15]; Eastern Europe, Northern Europe, Southern Europe, Western Europe.

^c ^Hospital size according to number of acute care beds; ≤ 300 beds, 301 – 600 beds, > 600 beds; information available for 294 hospitals.

^d^  ≤ / >  median defined as FTE ICN (internal staff and external staff) per 1,000 acute care hospital beds ≤ / > the median of all participating hospitals (3.72 FTE ICN/1,000 beds).

^e ^Differences between ≤ 300 beds / 301 – 600 beds / > 600 beds p < 0.05*.*

^f ^Differences between low/high HCE p < 0.05*.*

### Feedback on the HCAI situation and hand hygiene compliance

In almost all hospitals, healthcare workers (HCW) received feedback on HCAI (n = 106 more than twice a year, n = 61 twice a year, n = 115 once a year, n = 15 less than once a year). Of the 236 hospitals that performed ABHR consumption surveillance, 200 hospitals provided feedback at least once a year (n = 41 more than twice a year, n = 32 twice a year, n = 127 once a year), while in 35 hospitals feedback was given less than once a year and for one hospital information on the frequency of feedback was unavailable. Concerning direct hand hygiene compliance observations, 152 hospitals provided immediate feedback to the observed personnel and 131 hospitals provided a later summary feedback.

As shown in the Figure, IPC committees mainly received data on HCAI (n = 259; 87%) and the proportion of MDRO among HCAIs (n = 245; 83%) but less often on hand hygiene performance indicators. Feedback on MDRO among HCAIs was most often provided in larger hospitals (p = 0.012). IPC committees in hospitals with ICN rates above the European median received significantly more often feedback on hand hygiene compliance data compared to IPC committees in hospitals with ICN rates below the median (p = 0.039). Feedback on hand hygiene performance (ABHR consumption and/or hand hygiene compliance) was significantly more often provided in countries with high HCE (p = 0.042). No feedback was given to IPC committees in 23 (8%) hospitals.

**Figure:**
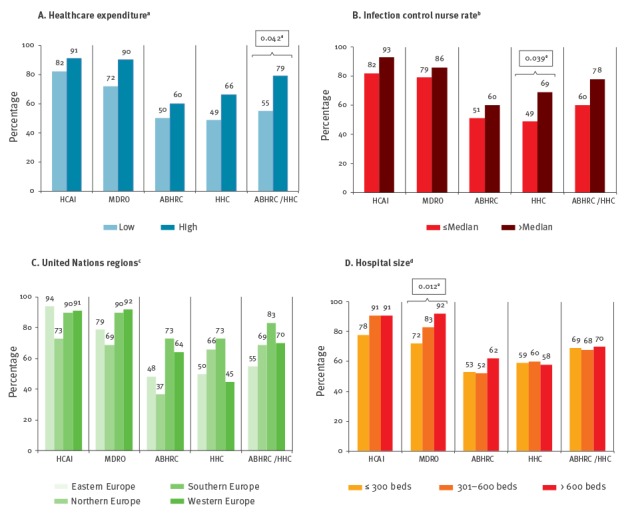
Feedback of surveillance data to the infection control committees in European acute care hospitals with established healthcare-associated infection surveillance, stratified by healthcare expenditure, infection control nurse rate, United Nation regions and hospital size – The Prevention of Hospital Infection by Intervention and Training (PROHIBIT) survey, 2011–2012 (n = 297 hospitals)

ABHRC: alcohol-based hand rub consumption; ABHRC/HHC: ABHRC and/or hand hygiene compliance; FTE: full time equivalent; HCAI: healthcare-associated infections; HCE: healthcare expenditure; HHC: hand hygiene compliance; ICN: infection control nurse; MDRO: multidrug-resistant organisms.

^a^ Low/high HCE defined as the share of the gross domestic product  ≤ / > the European mean in 2010 (9%) [16]; low HCE (n = 127), high HCE (n = 170).

^b^ FTE ICN (internal staff and external staff) per 1,000 acute care hospital beds ≤ / > the median of all participating hospitals (3.72 FTE ICN/1,000 beds); ‘≤ median’ (n = 150), ‘> median’ (n = 147).

^c^ Geographical regions according to United Nations grouping [15]; Eastern Europe (n = 82), Northern Europe (n = 70), Southern Europe (n = 81), Western Europe (n = 64).

^d^ Hospital size according to number of acute care beds; ‘≤ 300 beds’ (n = 87), ‘301–600 beds’ (n = 109), ‘> 600 beds’ (n = 98); information available for 294 hospitals.

^e^ P values were calculated by logistic regression using generalised estimating equations, which account for cluster effects by country.

## Discussion

To our knowledge, the data of the PROHIBIT survey offer the first broad analysis of HCAI and MDRO surveillance activities in European acute care hospitals. The findings show that content and methods of surveillance and the role of feedback vary widely across Europe. Hospitals focused more frequently on the surveillance of outcome indicators as BSI and SSI than on PN, CDI or UTI. This may be due to numerous success stories of BSI and SSI preventability, which raised hospitals’ awareness towards these two infection types [17-19].

Fifty-eight per cent of hospitals with SSI surveillance reported to have PDS in place. Such additional surveillance as described by Woelber et al. in 2016, partly prevents under-reporting of SSI in Europe [20]. The finding that HPRO is the most common indicator procedure for SSI surveillance corresponds to the results of ECDC’s HAI-Net surveillance of SSI with HPRO being the most frequently reported type of surgery, representing 33% of all operations in 2010–11 [21].

Nevertheless, successful preventability of HCAIs such as PN and UTI has also been described [22,23] and data of the PPS from 2011–12 indicate that respiratory tract infections and UTI are common HCAIs all over Europe. Interestingly, UTI surveillance was significantly more frequently reported in countries with low HCE. Data of the PPS for countries with high HCE however, showed frequencies of UTIs up to 31%, indicating that patients hospitalised in such countries are also at risk for this type of HCAI [1].

Resources are limited; and thus, priorities must be made in HCAI surveillance, even if a broad surveillance strategy including process and outcome indicators is considered helpful to tailor intervention activities for HCAI prevention. Prospective hospital-wide HCAI surveillance is resource-intensive, and in this sense, it was surprising to find that the proportion of hospitals with hospital-wide PN and UTI surveillance was significantly higher in hospitals from countries with low HCE compared to hospitals from countries with high HCE. Generally, hospital-wide surveillance of HCAI is time-consuming and repeated PPSs on HCAI or an automated surveillance linking administrative data and clinical databases including microbiology may be a better approach.

Large healthcare-associated outbreaks of CDI in the first decade of 2000 sparked increased awareness of CDI prevention in Europe, and resulted in European guidelines on CDI prevention in 2008, recommending CDI surveillance [24]. Data of our survey showed that fewer hospitals in countries with low HCE established CDI surveillance, indicating that surveillance activities as a whole may be influenced by financial constraints in Europe. The low level of CDI surveillance activities in these hospitals may be due to absent national CDI surveillance systems, but also to a lack of diagnostic testing and missing awareness as a consequence [1,25]. The high number of hospitals performing CDI surveillance in Northern Europe can be seen as a consequence of public reporting on CDI in the United Kingdom (UK). The new European protocol of CDI surveillance for acute care hospitals, which was developed in 2013, offers a standardised cross-country surveillance, with the option of integrating clinical and molecular data, and can contribute to enhanced monitoring of CDI in all parts of Europe [26].

Although low, still 10% of hospitals in Europe, and nearly 20% of hospitals in Eastern Europe, collect HCAI data by ward personnel only. This method of case finding can be interpreted as a more passive rather than active surveillance, with potential bias of under-reporting. As recommended by the Association for Professionals in Infection Control and Epidemiology (APIC), appropriate education to apply infection surveillance definitions or to perform detailed risk factor collection is indispensable [27].

In addition to the surveillance of outcomes, many hospitals assess data on hand hygiene performance indicators. Monitoring process indicators and assessment of adherence to IPC measures such as hand hygiene observation, enables hospitals to identify gaps and improve adherence to IPC measures more promptly than by focusing on outcome data alone. Interestingly, our study identified a discrepancy between a relatively high number of hospitals monitoring ABHR consumption and a relatively low number of hospitals giving feedback on ABHR consumption data to their IPC committees. This may be due the fact that many hospitals start surveillance activities focusing on outcome indicators and still work on the feedback of these indicators rather on reporting ABHR. In addition, ABHR data are often collected yearly, and thus, may be reported less frequently to IPC committees. On the other hand, process indicators are better candidates to be used for a realistic target-setting both at ward and hospital level. Reference data on these indicators facilitate inter-hospital comparison to support improving their processes [28]. Europe-wide surveys as the ECDC-PPS or PROHIBIT [1,14] already offer reference data on factors such as IPC personnel or isolation capacities and future projects may generate more, possibly stratified reference data for relevant structural and process IPC parameters.

In order to alter their behaviour in HCAI prevention HCWs have to be aware of the problem of HCAI in their setting. Data of our survey indicate that HCWs do receive feedback on HCAI rates in order to raise awareness. However, more research is needed to explore how surveillance data are communicated and perceived, and how this process can be further optimised. Feedback of data may be combined with behaviourally informed approaches such as the setting of long-term goals and encouraging involvement/participation of HCWs for creating local ownership and reflection on achievements and further activities.

Since successful implementation of IPC measures requires the participation of HCWs and other stakeholders, feedback to members of the IPC committee is essential. Especially in smaller hospitals, feedback is not always established yet. In which way the size of a hospital influences feedback of MDRO data to hospitals’ stakeholders cannot be fully answered. It can be speculated that larger hospitals see more MDROs, and thus, data are perceived more relevant, particularly because they care more frequently for patients with severe and/or chronic diseases.

In the future, all hospitals’ IPC committees should be encouraged to work with MDRO data in order to address supporting organisational factors such as leadership support and communication in MDRO transmission prevention and antibiotic stewardship programmes [29,30].

The current survey gives insight into established surveillance activities of European hospitals. However, there are some limitations:

Participation in the survey was voluntary, and thus, based mainly on hospitals’ interest rather than on a randomised sampling process. Therefore, the data may have overestimated surveillance activities in European hospitals. A randomly selected sample would have improved representation of hospitals in Europe. However, the questionnaire could not have been imposed on hospitals, and thus, data quality and the number of participating hospitals might have been lower.

The UN geographical regions are not homogeneous in terms of GDP, healthcare organisation and culture. However, by also reporting data in reference to countries’ HCE, we tried to take into account such heterogeneity.

Our findings show that objectives and methods of surveillance vary across Europe. Some outcome indicators, such as BSI, SSI and MRSA, seem to receive considerably more attention than others that are equally important, such as PN, UTI or CDI.

Hospitals’ IPC committees mainly receive data on outcome indicators as HCAI and MDRO, but less often on process indicators as hand hygiene performance indicators.

In order to better address prevention of HCAI and antimicrobial resistance in Europe surveillance should be further improved by targeting all relevant HCAI and MDRO and providing active surveillance by trained personnel. To what extent surveillance of process indicators prevent HCAI must be further analysed. In addition, the role of feedback and behaviourally informed approaches should be explored in more detail.

## Notes

**The PROHIBIT study group:** Pittet Didier, Zingg Walter, Sax Hugo, Gastmeier Petra, Hansen Sonja, Grundmann Hajo, van Benthem Birgit, van der Kooi Tjallie, Dettenkofer Markus, Martin Maria, Richet Hervé, Szilágyi Emese, Heczko Piotr, Holmes Alison, Kyratsis Yannis, Ahmad Raheelah, Allegranzi Benedetta, Magiorakos Anna-Pelagia, Cookson Barry, Wu Albert.
